# 4-Chloro­anilinium tetra­fluoro­borate 18-crown-6 clathrate

**DOI:** 10.1107/S1600536812006216

**Published:** 2012-02-17

**Authors:** Jie Xu, Min-Min Zhao

**Affiliations:** a Ordered Matter Science Research Center, College of Chemistry and Chemical Engineering, Southeast University, Nanjing 210096, People’s Republic of China

## Abstract

In the title compound, C_6_H_7_ClN^+^·BF_4_^−^·C_12_H_24_O_6_, the complete cation is generated by crystallographic mirror symmetry, with two C atoms and the N and Cl atoms lying on the mirror plane. The complete crown ether is also generated by mirror symmetry, as is the anion (in which the B and two F atoms lie on the mirror plane). The –NH_3_^+^ group of the cation inserts into the crown-ether ring and forms bifurcated N—H⋯(O,O) hydrogen bonds. The H atoms of the –NH_3_^+^ group were modelled as disordered across the mirror plane.

## Related literature

For the ferroelectric properties of related compounds, see: Fu *et al.* (2011 ▶)
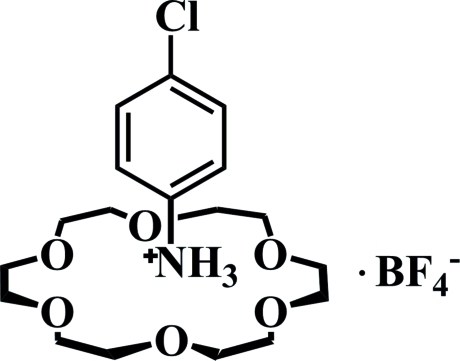


## Experimental

### 

#### Crystal data

C_6_H_7_ClN^+^·BF_4_^−^·C_12_H_24_O_6_
*M* *_r_* = 479.70
Orthorhombic, 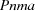

*a* = 15.619 (3) Å
*b* = 11.374 (2) Å
*c* = 12.956 (3) Å
*V* = 2301.6 (8) Å^3^
*Z* = 4
Mo *K*α radiation
μ = 0.23 mm^−1^
*T* = 298 K
0.10 × 0.03 × 0.03 mm

#### Data collection

Rigaku Mercury2 CCD diffractometer
Absorption correction: multi-scan (*CrystalClear*; Rigaku, 2005 ▶) *T*_min_ = 0.910, *T*_max_ = 1.000
22855 measured reflections
2772 independent reflections
1466 reflections with *I* > 2σ(*I*)
*R*_int_ = 0.112

#### Refinement

*R*[*F* ^2^ > 2σ(*F* ^2^)] = 0.068
*wR*(*F* ^2^) = 0.176
*S* = 1.04
2772 reflections
155 parameters
H-atom parameters constrained
Δρ_max_ = 0.24 e Å^−3^
Δρ_min_ = −0.26 e Å^−3^

### 

Data collection: *CrystalClear* (Rigaku, 2005 ▶); cell refinement: *CrystalClear*; data reduction: *CrystalClear*; program(s) used to solve structure: *SHELXS97* (Sheldrick, 2008 ▶); program(s) used to refine structure: *SHELXL97* (Sheldrick, 2008 ▶); molecular graphics: *SHELXTL* (Sheldrick, 2008 ▶); software used to prepare material for publication: *SHELXTL*.

## Supplementary Material

Crystal structure: contains datablock(s) I, global. DOI: 10.1107/S1600536812006216/hb6580sup1.cif

Structure factors: contains datablock(s) I. DOI: 10.1107/S1600536812006216/hb6580Isup2.hkl

Supplementary material file. DOI: 10.1107/S1600536812006216/hb6580Isup3.cml

Additional supplementary materials:  crystallographic information; 3D view; checkCIF report

## Figures and Tables

**Table 1:** Hydrogen-bond geometry (Å, °)

*D*—H⋯*A*	*D*—H	H⋯*A*	*D*⋯*A*	*D*—H⋯*A*
N1—H1*A*⋯O2	0.89	2.12	2.897 (3)	146
N1—H1*A*⋯O3	0.89	2.24	2.927 (3)	134
N1—H1*B*⋯O3^i^	0.89	2.25	2.927 (3)	133
N1—H1*B*⋯O4	0.89	2.10	2.891 (4)	147
N1—H1*C*⋯O1	0.89	2.21	2.865 (4)	130
N1—H1*C*⋯O2^i^	0.89	2.10	2.897 (3)	148

Symmetry code: (i) 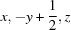.

**Fig. 1.**
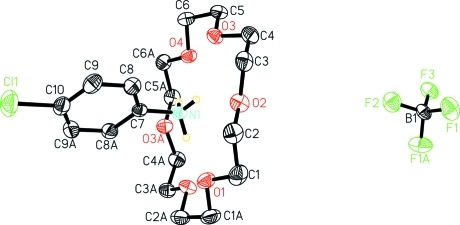
A view of the title compound with displacement ellipsoids drawn at the 30% probability level. H atoms have been omitted for clarity.

**Fig. 2.**
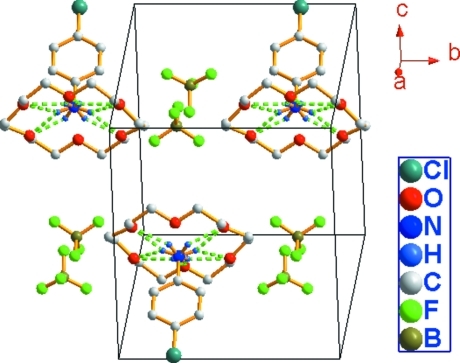
The crystal packing of the title compound, showing the H-bonding interactions. H atoms not involved in hydrogen bonding (dashed line) have been omitted for clarity.

## supplementary crystallographic information

### Comment

With the purpose of obtaining phase transition crystals of amino compounds, various amines have been studied and we have elaborated a series of new materials with this organic molecules (Fu *et al.* 2011). In this study, we describe the crystal structure of the title compound.

The asymmetric unit is composed of one organic cation, one BF_4_^-^ anion and one crown ether (Fig.1). The 4-chloroanilium cation and macrocyclic ether mlecule are associated *vis* hydrogen bonding with the -NH_3_^+^ group forming bifurcated bonds with all six O atoms of 18-crown-6 molecule. Despite the disorder in the amino group, it is clear that in each orientation the cation forms three bifurcated hydrogen bonds. These H-bonding interactions link the cation and 18-crown-6 ether molecule into a 1:1 complex, [(C_6_H_7_ClN).(18-rown-6)]^+^ (Table 1 and Fig.2).

### Experimental

18-Crown-6 (3 mmol), HBF_4_ (5 mmol) and the organic amine (3 mmol) were dissolved in water/EtOH (1:1 *v*/*v*) solution. The solvent was slowly evaporated in air affording colourless block-shaped crystals of the title compound.

The dielectric constant of title compound as a function of temperature indicates that the permittivity is basically temperature-independent, suggesting that this compound should be not a real ferroelectrics or there may be no distinct phase transition occurred within the measured temperature range. Similarly, below the melting point (405 K) of the compound, the dielectric constant as a function of temperature also goes smoothly, and there is no dielectric anomaly observed (dielectric constant ranging from 4.1 to 8.1).

### Refinement

All H atoms attached to C atoms were fixed geometrically and treated as riding with C–H = 0.93 Å (Caromatic) or 0.97 Å (Cmethylene).

The positional parameters of the H atoms (N1) were intially refined freely, subsequently restrained using a distance of N–H = 0.89 (2) Å, and in the final refinements treated in riding motion of their parent nitrogen atom with *U_iso_*(H)=1.5*U_eq_*(N).

### Crystal data

**Table:**

C_6_H_7_ClN^+^·BF_4_^−^·C_12_H_24_O_6_	*F*(000) = 1008
*M**_r_* = 479.70	*D*_x_ = 1.384 Mg m^−^^3^
Orthorhombic, *P**n**m**a*	Mo *K*α radiation, λ = 0.71073 Å
Hall symbol: -P 2ac 2n	Cell parameters from 2772 reflections
*a* = 15.619 (3) Å	θ = 3.1–27.5°
*b* = 11.374 (2) Å	µ = 0.23 mm^−^^1^
*c* = 12.956 (3) Å	*T* = 298 K
*V* = 2301.6 (8) Å^3^	Block, colorless
*Z* = 4	0.10 × 0.03 × 0.03 mm

### Data collection

**Table:**

Rigaku Mercury2 CCD diffractometer	2772 independent reflections
Radiation source: fine-focus sealed tube	1466 reflections with *I* > 2σ(*I*)
Graphite monochromator	*R*_int_ = 0.112
Detector resolution: 13.6612 pixels mm^-1^	θ_max_ = 27.5°, θ_min_ = 3.1°
CCD profile fitting scans	*h* = −20→20
Absorption correction: multi-scan (*CrystalClear*; Rigaku, 2005)	*k* = −14→14
*T*_min_ = 0.910, *T*_max_ = 1.000	*l* = −16→16
22855 measured reflections	

### Refinement

**Table:**

Refinement on *F*^2^	Primary atom site location: structure-invariant direct methods
Least-squares matrix: full	Secondary atom site location: difference Fourier map
*R*[*F*^2^ > 2σ(*F*^2^)] = 0.068	Hydrogen site location: inferred from neighbouring sites
*wR*(*F*^2^) = 0.176	H-atom parameters constrained
*S* = 1.04	*w* = 1/[σ^2^(*F*_o_^2^) + (0.0645*P*)^2^ + 0.8791*P*] where *P* = (*F*_o_^2^ + 2*F*_c_^2^)/3
2772 reflections	(Δ/σ)_max_ < 0.001
155 parameters	Δρ_max_ = 0.24 e Å^−^^3^
0 restraints	Δρ_min_ = −0.26 e Å^−^^3^

### Special details

**Table:**

Geometry. All esds (except the esd in the dihedral angle between two l.s. planes) are estimated using the full covariance matrix. The cell esds are taken into account individually in the estimation of esds in distances, angles and torsion angles; correlations between esds in cell parameters are only used when they are defined by crystal symmetry. An approximate (isotropic) treatment of cell esds is used for estimating esds involving l.s. planes.
Refinement. Refinement of F^2^ against ALL reflections. The weighted R-factor wR and goodness of fit S are based on F^2^, conventional R-factors R are based on F, with F set to zero for negative F^2^. The threshold expression of F^2^ > 2sigma(F^2^) is used only for calculating R-factors(gt) etc. and is not relevant to the choice of reflections for refinement. R-factors based on F^2^ are statistically about twice as large as those based on F, and R- factors based on ALL data will be even larger.

### Fractional atomic coordinates and isotropic or equivalent isotropic displacement parameters (Å^2^)

**Table:**

	*x*	*y*	*z*	*U*_iso_*/*U*_eq_	Occ. (<1)
Cl1	1.00491 (9)	0.2500	0.01849 (12)	0.1019 (6)	
O4	0.54013 (18)	0.2500	0.1542 (2)	0.0496 (7)	
O2	0.69794 (13)	0.04251 (17)	0.40917 (15)	0.0561 (6)	
N1	0.6932 (2)	0.2500	0.2796 (2)	0.0430 (8)	
H1A	0.6806	0.1765	0.2975	0.064*	0.50
H1B	0.6495	0.2811	0.2451	0.064*	0.50
H1C	0.7033	0.2924	0.3360	0.064*	0.50
O1	0.78267 (19)	0.2500	0.4727 (2)	0.0574 (8)	
O3	0.60482 (13)	0.03256 (17)	0.22269 (15)	0.0518 (6)	
C7	0.7692 (2)	0.2500	0.2142 (3)	0.0397 (9)	
C5	0.5276 (2)	0.0445 (3)	0.1647 (2)	0.0555 (8)	
H5A	0.4797	0.0563	0.2110	0.067*	
H5B	0.5173	−0.0266	0.1251	0.067*	
C8	0.8049 (2)	0.1449 (3)	0.1844 (2)	0.0533 (8)	
H8A	0.7802	0.0743	0.2048	0.064*	
C3	0.6806 (2)	−0.0649 (3)	0.3568 (3)	0.0588 (9)	
H3A	0.6778	−0.1291	0.4060	0.071*	
H3B	0.7259	−0.0816	0.3077	0.071*	
C6	0.5358 (2)	0.1464 (3)	0.0940 (2)	0.0548 (8)	
H6A	0.5871	0.1386	0.0525	0.066*	
H6B	0.4868	0.1498	0.0481	0.066*	
C4	0.5972 (2)	−0.0534 (3)	0.3018 (2)	0.0600 (9)	
H4A	0.5813	−0.1285	0.2719	0.072*	
H4B	0.5529	−0.0304	0.3501	0.072*	
C2	0.7766 (2)	0.0415 (3)	0.4644 (3)	0.0642 (9)	
H2A	0.8242	0.0441	0.4164	0.077*	
H2B	0.7811	−0.0300	0.5048	0.077*	
C10	0.9133 (3)	0.2500	0.0942 (3)	0.0620 (13)	
C1	0.7793 (2)	0.1455 (3)	0.5335 (3)	0.0698 (10)	
H1D	0.7287	0.1468	0.5770	0.084*	
H1E	0.8293	0.1412	0.5777	0.084*	
C9	0.8773 (2)	0.1450 (3)	0.1244 (2)	0.0633 (9)	
H9A	0.9020	0.0743	0.1042	0.076*	
F3	0.02293 (17)	0.2500	0.6646 (2)	0.0761 (8)	
F2	0.16106 (19)	0.2500	0.6182 (3)	0.0941 (10)	
F1	0.12000 (15)	0.1503 (2)	0.7597 (2)	0.1066 (8)	
B1	0.1052 (3)	0.2500	0.7028 (5)	0.0574 (14)	

### Atomic displacement parameters (Å^2^)

**Table:**

	*U*^11^	*U*^22^	*U*^33^	*U*^12^	*U*^13^	*U*^23^
Cl1	0.0634 (9)	0.1501 (15)	0.0924 (11)	0.000	0.0263 (8)	0.000
O4	0.0613 (18)	0.0453 (17)	0.0421 (16)	0.000	−0.0095 (13)	0.000
O2	0.0553 (13)	0.0470 (13)	0.0660 (14)	0.0079 (10)	−0.0087 (11)	0.0063 (10)
N1	0.046 (2)	0.0414 (18)	0.0417 (18)	0.000	−0.0073 (15)	0.000
O1	0.068 (2)	0.063 (2)	0.0418 (16)	0.000	−0.0087 (14)	0.000
O3	0.0574 (13)	0.0414 (11)	0.0567 (12)	−0.0060 (9)	−0.0027 (10)	0.0042 (10)
C7	0.041 (2)	0.044 (2)	0.034 (2)	0.000	−0.0091 (17)	0.000
C5	0.057 (2)	0.0509 (19)	0.058 (2)	−0.0101 (15)	−0.0079 (15)	−0.0093 (15)
C8	0.062 (2)	0.0459 (18)	0.0525 (18)	0.0046 (15)	0.0009 (16)	0.0024 (14)
C3	0.072 (2)	0.0397 (18)	0.065 (2)	0.0078 (15)	0.0068 (18)	0.0125 (16)
C6	0.061 (2)	0.056 (2)	0.0470 (17)	−0.0014 (15)	−0.0079 (14)	−0.0113 (15)
C4	0.071 (2)	0.0425 (19)	0.067 (2)	−0.0060 (15)	0.0026 (18)	0.0035 (16)
C2	0.061 (2)	0.067 (2)	0.064 (2)	0.0090 (17)	−0.0110 (17)	0.0142 (18)
C10	0.044 (3)	0.098 (4)	0.044 (3)	0.000	−0.005 (2)	0.000
C1	0.070 (2)	0.090 (3)	0.0490 (19)	0.0054 (19)	−0.0131 (17)	0.017 (2)
C9	0.065 (2)	0.070 (2)	0.0550 (19)	0.0169 (18)	0.0026 (17)	−0.0039 (18)
F3	0.0625 (18)	0.0647 (18)	0.101 (2)	0.000	−0.0047 (15)	0.000
F2	0.075 (2)	0.102 (2)	0.105 (2)	0.000	0.0133 (18)	0.000
F1	0.0994 (17)	0.0917 (17)	0.1288 (19)	0.0004 (13)	−0.0163 (15)	0.0429 (16)
B1	0.049 (3)	0.043 (3)	0.080 (4)	0.000	0.006 (3)	0.000

### Geometric parameters (Å, º)

**Table:**

Cl1—C10	1.735 (5)	C3—C4	1.490 (4)
O4—C6	1.415 (3)	C3—H3A	0.9700
O4—C6^i^	1.415 (3)	C3—H3B	0.9700
O2—C2	1.421 (4)	C6—H6A	0.9700
O2—C3	1.424 (4)	C6—H6B	0.9700
N1—C7	1.457 (5)	C4—H4A	0.9700
N1—H1A	0.8900	C4—H4B	0.9700
N1—H1B	0.8900	C2—C1	1.484 (4)
N1—H1C	0.8900	C2—H2A	0.9700
O1—C1^i^	1.427 (3)	C2—H2B	0.9700
O1—C1	1.427 (3)	C10—C9^i^	1.376 (4)
O3—C4	1.422 (3)	C10—C9	1.376 (4)
O3—C5	1.427 (3)	C1—H1D	0.9700
C7—C8	1.375 (3)	C1—H1E	0.9700
C7—C8^i^	1.375 (3)	C9—H9A	0.9300
C5—C6	1.482 (4)	F3—B1	1.377 (6)
C5—H5A	0.9700	F2—B1	1.401 (6)
C5—H5B	0.9700	F1—B1	1.372 (4)
C8—C9	1.371 (4)	B1—F1^i^	1.372 (4)
C8—H8A	0.9300		
			
C6—O4—C6^i^	112.8 (3)	C5—C6—H6B	110.0
C2—O2—C3	113.4 (2)	H6A—C6—H6B	108.4
C7—N1—H1A	109.5	O3—C4—C3	109.4 (2)
C7—N1—H1B	109.5	O3—C4—H4A	109.8
H1A—N1—H1B	109.5	C3—C4—H4A	109.8
C7—N1—H1C	109.5	O3—C4—H4B	109.8
H1A—N1—H1C	109.5	C3—C4—H4B	109.8
H1B—N1—H1C	109.5	H4A—C4—H4B	108.2
C1^i^—O1—C1	112.8 (3)	O2—C2—C1	108.8 (3)
C4—O3—C5	112.0 (2)	O2—C2—H2A	109.9
C8—C7—C8^i^	120.8 (4)	C1—C2—H2A	109.9
C8—C7—N1	119.62 (19)	O2—C2—H2B	109.9
C8^i^—C7—N1	119.62 (19)	C1—C2—H2B	109.9
O3—C5—C6	109.1 (2)	H2A—C2—H2B	108.3
O3—C5—H5A	109.9	C9^i^—C10—C9	120.3 (4)
C6—C5—H5A	109.9	C9^i^—C10—Cl1	119.8 (2)
O3—C5—H5B	109.9	C9—C10—Cl1	119.8 (2)
C6—C5—H5B	109.9	O1—C1—C2	109.3 (2)
H5A—C5—H5B	108.3	O1—C1—H1D	109.8
C9—C8—C7	119.6 (3)	C2—C1—H1D	109.8
C9—C8—H8A	120.2	O1—C1—H1E	109.8
C7—C8—H8A	120.2	C2—C1—H1E	109.8
O2—C3—C4	108.6 (2)	H1D—C1—H1E	108.3
O2—C3—H3A	110.0	C8—C9—C10	119.9 (3)
C4—C3—H3A	110.0	C8—C9—H9A	120.1
O2—C3—H3B	110.0	C10—C9—H9A	120.1
C4—C3—H3B	110.0	F1^i^—B1—F1	111.5 (5)
H3A—C3—H3B	108.3	F1^i^—B1—F3	110.5 (3)
O4—C6—C5	108.4 (2)	F1—B1—F3	110.5 (3)
O4—C6—H6A	110.0	F1^i^—B1—F2	108.4 (3)
C5—C6—H6A	110.0	F1—B1—F2	108.4 (3)
O4—C6—H6B	110.0	F3—B1—F2	107.5 (4)

Symmetry code: (i) *x*, −*y*+1/2, *z*.

### Hydrogen-bond geometry (Å, º)

**Table:**

*D*—H···*A*	*D*—H	H···*A*	*D*···*A*	*D*—H···*A*
N1—H1*A*···O2	0.89	2.12	2.897 (3)	146
N1—H1*A*···O3	0.89	2.24	2.927 (3)	134
N1—H1*B*···O3^i^	0.89	2.25	2.927 (3)	133
N1—H1*B*···O4	0.89	2.10	2.891 (4)	147
N1—H1*C*···O1	0.89	2.21	2.865 (4)	130
N1—H1*C*···O2^i^	0.89	2.10	2.897 (3)	148

Symmetry code: (i) *x*, −*y*+1/2, *z*.
